# Loss of PRCD alters number and packaging density of rhodopsin in rod photoreceptor disc membranes

**DOI:** 10.1038/s41598-020-74628-2

**Published:** 2020-10-21

**Authors:** Emily R. Sechrest, Joseph Murphy, Subhadip Senapati, Andrew F. X. Goldberg, Paul S.-H. Park, Saravanan Kolandaivelu

**Affiliations:** 1 Department of Pharmaceutical Sciences, One Medical Center Drive, West Virginia University, Morgantown, WV 26506-9193 USA; 2 Department of Ophthalmology and Visual Sciences, Eye Institute, One Medical Center Drive, West Virginia University, Morgantown, WV 26506-9193 USA; 3 Department of Biochemistry, One Medical Center Drive, West Virginia University, Morgantown, WV 26506-9193 USA; 4 Department of Ophthalmology and Visual Sciences, Case Western Reserve University, Cleveland, OH 44106 USA; 5 Eye Research Institute, Oakland University, Rochester, MI 48309 USA

**Keywords:** Retina, Neurodegeneration, Biochemistry

## Abstract

Progressive rod-cone degeneration (PRCD) is a small protein localized to photoreceptor outer segment (OS) disc membranes. Several mutations in PRCD are linked to retinitis pigmentosa (RP) in canines and humans, and while recent studies have established that PRCD is required for high fidelity disc morphogenesis, its precise role in this process remains a mystery. To better understand the part which PRCD plays in disease progression as well as its contribution to photoreceptor OS disc morphogenesis, we generated a *Prcd*-KO animal model using CRISPR/Cas9. Loss of PRCD from the retina results in reduced visual function accompanied by slow rod photoreceptor degeneration. We observed a significant decrease in rhodopsin levels in *Prcd*-KO retina prior to photoreceptor degeneration. Furthermore, ultrastructural analysis demonstrates that rod photoreceptors lacking PRCD display disoriented and dysmorphic OS disc membranes. Strikingly, atomic force microscopy reveals that many disc membranes in *Prcd*-KO rod photoreceptor neurons are irregular, containing fewer rhodopsin molecules and decreased rhodopsin packing density compared to wild-type discs. This study strongly suggests an important role for PRCD in regulation of rhodopsin incorporation and packaging density into disc membranes, a process which, when dysregulated, likely gives rise to the visual defects observed in patients with PRCD-associated RP.

## Introduction

Rod and cone photoreceptor neurons contain a distinct membrane-rich structure at the cilium known as the outer segment (OS). Each rod OS (ROS) is packed with over 1000 tightly stacked, double membranous discs which house phototransduction proteins required for normal photoreceptor function and structural stability^1–6^. In this study, we focus on progressive rod-cone degeneration (PRCD), a small 54 amino acid (53 amino acid in mice) protein which is specifically localized to OS disc membranes^7^. The most common mutation in PRCD is a C2Y mutation, which is linked to retinitis pigmentosa (RP) in humans and progressive retinal atrophy (PRA) in over 29 dog breeds^8–10^. RP and PRA are both hereditary retinal disorders which result in degeneration of rods followed by cones, leading eventually to complete blindness^11–13^. In canines and humans, PRCD-associated disease is characterized by progressive loss of vision and severe disorganization of OS disc membranes^10,14–16^.

Previous studies, including ours, have demonstrated that PRCD undergoes palmitoylation, a post-translational lipid modification. Loss of palmitoylation in PRCD-C2Y results in mislocalization of PRCD to the inner segment (IS), where it is rapidly degraded^17,18^. Additionally, PRCD has been shown to interact with rhodopsin, a highly abundant ROS protein which is responsible for initiation of phototransduction^18–20^. While the significance of this interaction is not well understood, many animal models containing mutations in rhodopsin have demonstrated the crucial role rhodopsin plays in ROS maintenance and disc morphogenesis^21–27^. Within the ROS disc membranes, rhodopsin forms oligomers which are further organized into densely packed nanoscale domains^28–30^. Atomic force microscopy (AFM) imaging can achieve nanoscale resolution adequate to visualize these nanodomains^31^. Strikingly, these AFM studies demonstrate that there are biological mechanisms in place to ensure the density of rhodopsin in ROS disc membranes is kept constant, suggesting that regulation of proper rhodopsin density is crucial for photoreceptor disc formation and health^32,33^.

Although PRCD’s specific role within the photoreceptor remains elusive, recent studies in a PRCD knockout mouse model have identified a requirement for PRCD in high fidelity disc morphogenesis. In this model, it is observed that discs do not flatten or form properly in the absence of PRCD. Consequently, membrane bulges containing rhodopsin appear to separate into extracellular vesicles and accrue within the interphotoreceptor space^34^. A second PRCD knockout mouse model has also shown accumulation of these vesicles and observed a reduced rate of phagocytosis of OS discs by the retinal pigment epithelium (RPE)^35^. Along with evidence of retinal degeneration in these models and structural defects exacerbated by microglia recruitment to clear these vesicles, it is clear that PRCD is required for proper OS maintenance and disc morphogenesis; however further studies are needed to clarify the precise mechanistic role which PRCD plays in these processes.

To further understand the interaction between PRCD and rhodopsin, as well as PRCD’s role in photoreceptor OS disc morphogenesis and maintenance, we utilized our *Prcd*-KO mouse model. In this study, we demonstrate that *Prcd*-KO retina display dramatic disorganization of the photoreceptor OS. Most importantly, using AFM imaging, we observe that loss of PRCD leads to decreased rhodopsin number and packaging density in ROS disc membranes, likely associated with the observed morphological changes in *Prcd*-KO retina.

## Results

### Generation of *Prcd*-KO mice using CRISPR/Cas9 genome editing

**Figure 1:**
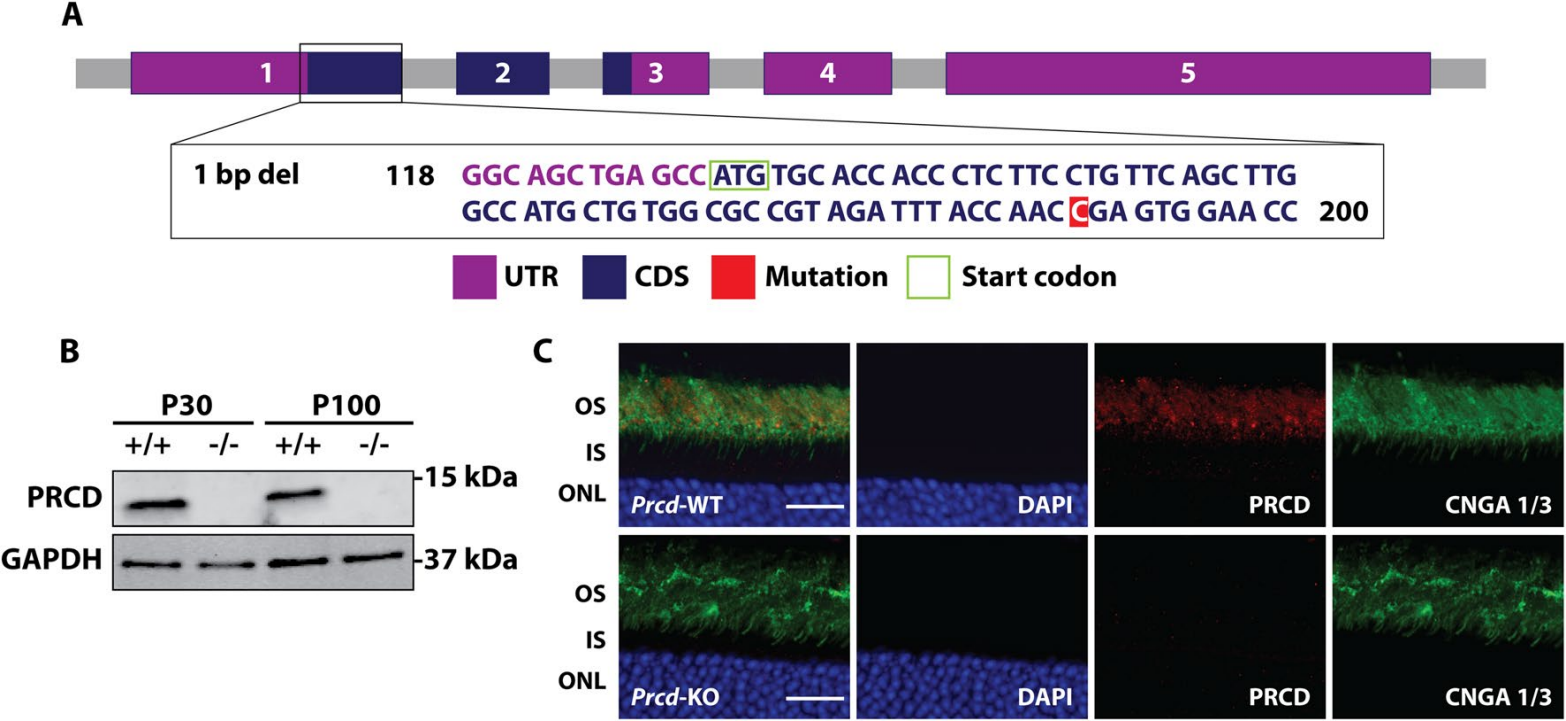
Generation and validation of *Prcd-*KO animal model. (**A**) Scheme demonstrating the generation of the *Prcd-*KO animal model using CRISPR/Cas9 technology. In this study, we used the 1738 founder line, which has a single base pair deletion in exon 1 of *Prcd*. UTR = untranslated region, CDS = coding sequence. (**B**) Immunoblot analysis shows loss of PRCD protein from *Prcd-*KO retinal lysate at postnatal (P) 30 and P100, whereas wild-type (WT) retinal lysates demonstrate PRCD immunoreactivity (n = 4). Please note that immunoblot data is cropped; full-length, raw data is available in Supplementary Fig. S2. (**C**) Immunofluorescent staining of P30 retinal cross-sections from WT and *Prcd-*KO mice, probing with antibodies against PRCD (red) and the OS marker cyclic nucleotide gated channel alpha-1 and alpha-3 (CNGA 1/3; green) to demonstrate proper localization of PRCD to the OS in WT retina and loss of PRCD from *Prcd-*KO retina (n = 3). Scale bar = 20 μm. All experiments were conducted with littermate controls.

To better understand the importance of PRCD in the maintenance and stability of the photoreceptor outer segment (OS), we generated a global *Prcd-*KO mouse model using CRISPR/Cas9 genome editing. Among 48 founder mice, we found 12 contained INDEL deletions in exon 1. For this study, we collected data from the 1738 founder, whose *Prcd* gene contains a single nucleotide deletion in exon 1 at cytosine 61 (c61x), resulting in a frameshift mutation (Fig. 1A and Supplementary Fig. S1). To rule out off-target effects, additional founder lines were tested, including mice with a 14 bp deletion (1734) and 71 bp deletion in *Prcd* (1748) (Supplementary Fig. S1). All lines exhibited similar phenotypes, were comparable in weight and development to wild-type (WT) littermates and are fertile with no detectable health issues. Western blot analysis demonstrated that lysate from *Prcd*-KO retina does not contain PRCD protein (Fig. 1B). Co-immunofluorescent labeling of retinal cross sections with antibodies against PRCD and the OS marker cyclic nucleotide gated channel A (CNGA) revealed expected PRCD OS localization in WT retina, whereas PRCD labeling was absent in *Prcd-*KO retinal sections (Fig. 1C). Altogether, these data validate the absence of PRCD protein in the *Prcd-*KO animal model used in this study.

### Loss of PRCD leads to progressive loss of visual function and slow photoreceptor degeneration

**Figure 2:**
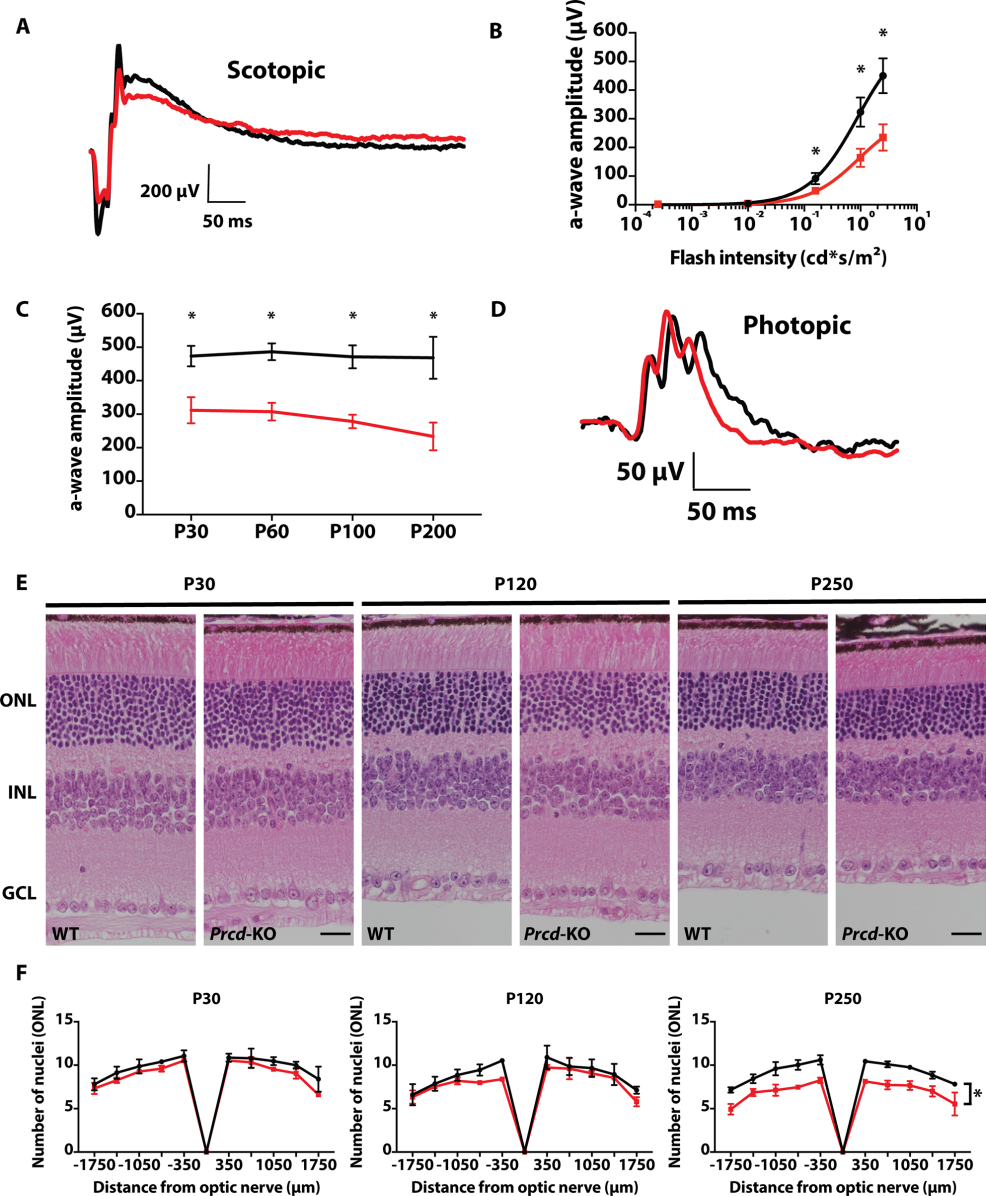
Reduced rod photoreceptor function and slow retinal degeneration in animals lacking *Prcd*. (**A**) Representative waveform from scotopic ERGs of WT (black) and *Prcd*-KO (red) animals at P30 (0.995 cd*s/m^2^). (**B**) Significant loss of rod photoreceptor function at higher light intensities (0.158, 0.995, and 2.5 cd*s/m^2^) compared with low light conditions (0.00025 and 0.001 cd*s/m^2^) (n = 4, data from B and C stats are unpaired two-tailed t-test; higher light intensities were statistically significant, *p < 0.01; Low light intensities were not significant). (**C**) Maximum a-wave amplitude of Prcd-KO animals at different ages compared to WT controls from P30 to P200. (**D**) Representative waveform from photopic (cone) ERGs of Prcd-KO and WT animals at P200 (7.9 cd*s/m^2^) (n = 4). (**E**) *Prcd*-KO and WT littermate control cross-sections stained with hematoxylin and eosin (H&E), imaged by light microscopy, at P30, P120, and P250. Scale bar = 20 μm. (**F**) Quantification of number of photoreceptor nuclei in the outer nuclear layer (ONL) from both *Prcd-*KO (red) and WT littermate controls (black), every 350 μm from the optic nerve, at P30, P120, and P250. Data are represented as mean (n = 3, unpaired two-tailed t-test; *p < 0.01).

To evaluate the visual response of rod and cone photoreceptor neurons, we assessed scotopic (rod) and photopic (cone) responses in *Prcd*-KO and WT littermates by electroretinography (ERG). A light-evoked ERG response is comprised of an “a-wave” (photoreceptor) and a “b-wave” (inner retinal neurons), which result from hyperpolarization of photoreceptors and depolarization of downstream retinal neurons, respectively^36^. At postnatal day (P) 30, *Prcd*-KO mice present with an approximate 30% reduction in rod response compared to WT littermate controls in bright light conditions (Fig. 2A). At low light intensities (0.00025 and 0.001 cd*s/m^2^), we were unable to identify any discernable differences in scotopic rod response between WT and *Prcd-*KO animals. However, at brighter flash intensities 0.158 (*p* = 0.009424), 0.995 (*p* = 0.001771), and 2.5 cd*s/m^2^ (*p* = 0.001363), rod a-waves were significantly reduced in *Prcd-*KO animals compared to WT littermate controls (Fig. 2B). Furthermore, we observed a slow decrease in visual response over time at various ages (P30, P60, P100, and P200); by P200, *Prcd*-KO animals demonstrate an approximate 50% reduction in rod photoresponse compared to WT littermate controls (Fig. 2C). In contrast, *Prcd*-KO photopic ERGs demonstrated no noticeable changes compared to WT responses at any tested ages or flash intensities (Fig. 2D). The evident impairment and gradual reduction in rod visual function of *Prcd*-KO animals suggests a possible defect in the morphology or survival of photoreceptor cells. We utilized hematoxylin and eosin (H&E) staining of WT and *Prcd*-KO retina at P30, P120, and P250 in order to analyze retinal morphology. No significant defects in retinal lamination or morphology were observed in P30 *Prcd*-KO mice (Fig. 2E). To determine if retinal degeneration was evident at P30, where we observed significant loss of rod function in bright light conditions, we counted photoreceptor nuclei at 10 different locations in the same H&E sections. No significant reduction in photoreceptor nuclei was apparent in retina lacking PRCD until after P120. At P250, we observed a significant reduction in photoreceptor nuclei, with approximately 23.8% fewer nuclei in *Prcd-*KO retina compared to WT (*p* = 0.001332; Fig. 2F). Based on these data, the early rod ERG defects observed in bright light conditions were likely not due to loss of rod photoreceptors.

### *Prcd-*KO retina exhibit reduced levels of rhodopsin and increased structural disorganization of rod OS as disease progresses

**Figure 3:**
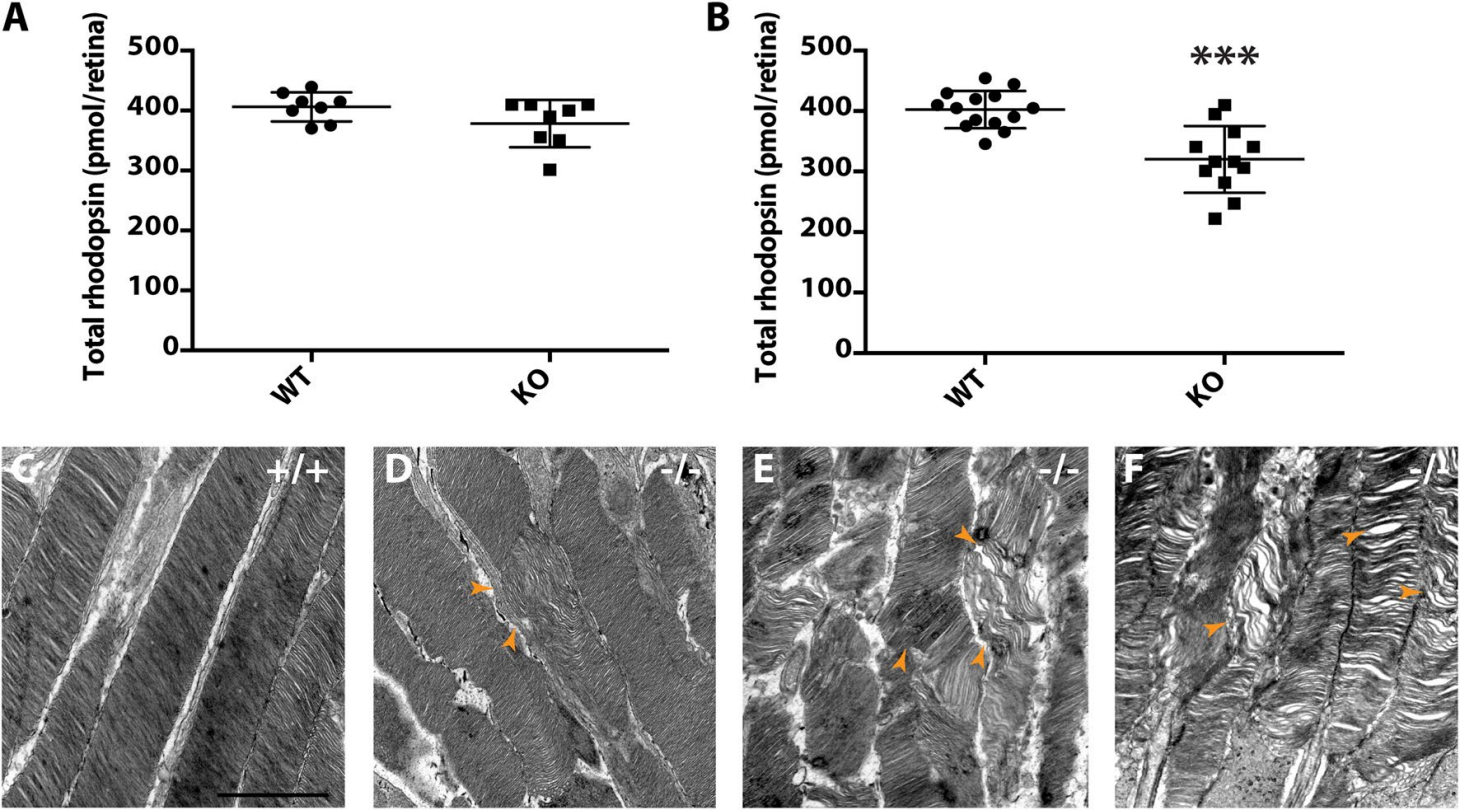
Reduced rhodopsin concentration and aberrant ultrastructure of photoreceptor OS during disease progression in *Prcd-*KO mice. (**A**) Quantification of total rhodopsin (pmol rhodopsin/retina) in WT and *Prcd*-KO retina at P30. (**B**) Quantification of total rhodopsin (pmol rhodopsin/retina) in WT and *Prcd*-KO retina at P120. Data is reported as the mean (unpaired, two-tailed t-test; ***p = .000072). (**C**) TEM image of the photoreceptor OS and well organized disc membranes at P120 (WT). (**D-F**) TEM ultrastructure of photoreceptor OS at various ages in *Prcd*-KO retina show the progressive disruption of OS structure from (**D**) P30, (**E**) P60, and (**F**) P120. Arrowheads indicate the defective and disorganized disc membranes. Scale bar = 500 nm.

As *Prcd*-KO retina do not undergo degeneration until after P120, it is possible that the observed defect in visual function is due to changes in major photoreceptor or disc-resident proteins, as PRCD is localized exclusively to the OS disc membrane. Therefore, we analyzed retinal lysate from WT and *Prcd*-KO mice at P30 and P100 by western blot and found no significant changes in any tested proteins (Supplementary Fig. S3). However, due to the reported interaction between rhodopsin and PRCD, we measured the concentration of rhodopsin in WT and *Prcd-*KO retina with spectrophotometric analysis, a technique which has been widely used to measure and calculate picomolar concentrations of rhodopsin in the retina^37,38^. At P30, total rhodopsin concentration was similar between *Prcd*-KO and WT retina (Fig. 3A). However, by P120, we observed a 20.4% decrease (*p* = 0.000072) in the amount of rhodopsin per *Prcd*-KO retina compared to age-matched WT controls (Fig. 3B). Despite the observed decrease in rhodopsin protein at P120, rhodopsin mRNA levels were comparable between WT and *Prcd*-KO animals at P30 and P120 (Supplementary Fig. S4). Furthermore, no mislocalization of rhodopsin is evident at P120 in *Prcd-*KO retinal sections when analyzed by immunofluorescent staining (Supplementary Fig. S5).

To gain further insight into the observed reduction in rod photoreceptor function at P30, we investigated photoreceptor ultrastructure at various ages (P30, P60, and P120) using transmission electron microscopy (TEM). At the earliest tested age (P30), many *Prcd*-KO rod photoreceptors contained discs that were disoriented and vertically aligned, although rod OS had an overall normal shape (Fig. 3D; arrowhead). Ultrastructural analysis of *Prcd-*KO rods at P60 showed the OS layer to be disorganized compared to WT. Outer segments had irregular diameters, included frequent examples of overgrown and misoriented disc membranes, and occasionally shed OS fragments and whorls of membrane packets into the OS layer. Closer inspection revealed a distinct population of *Prcd*-KO rods containing small packets of overgrown discs (10–40 discs), which were aligned abnormally, having grown along the long axis of the photoreceptors (Fig. 3E and Supplementary Fig. S6B–E). Although these defects were observed in *Prcd-*KO rods, there were still many rods which were comparable to WT, with no apparent differences in gross OS ultrastructure and contained nicely stacked, correctly sized, and properly aligned disc membranes (Supplementary Fig. S6A,B,F). Additionally, our evaluation of *Prcd*-KO retina ultrastructure found no apparent difference in the connecting cilium, inner segment (IS), mitochondria, or adjacent RPE layer compared to WT littermates. With increased age, *Prcd-*KO mice showed an increased frequency of defective photoreceptor cells with incorrectly stacked discs, further disorganization of ROS, and more space between discs at P120 compared to age-matched WT (Fig. 3C,F).

### Disc membranes in *Prcd*-KO rod photoreceptors contain reduced packaging density and number of rhodopsin molecules

**Figure 4:**
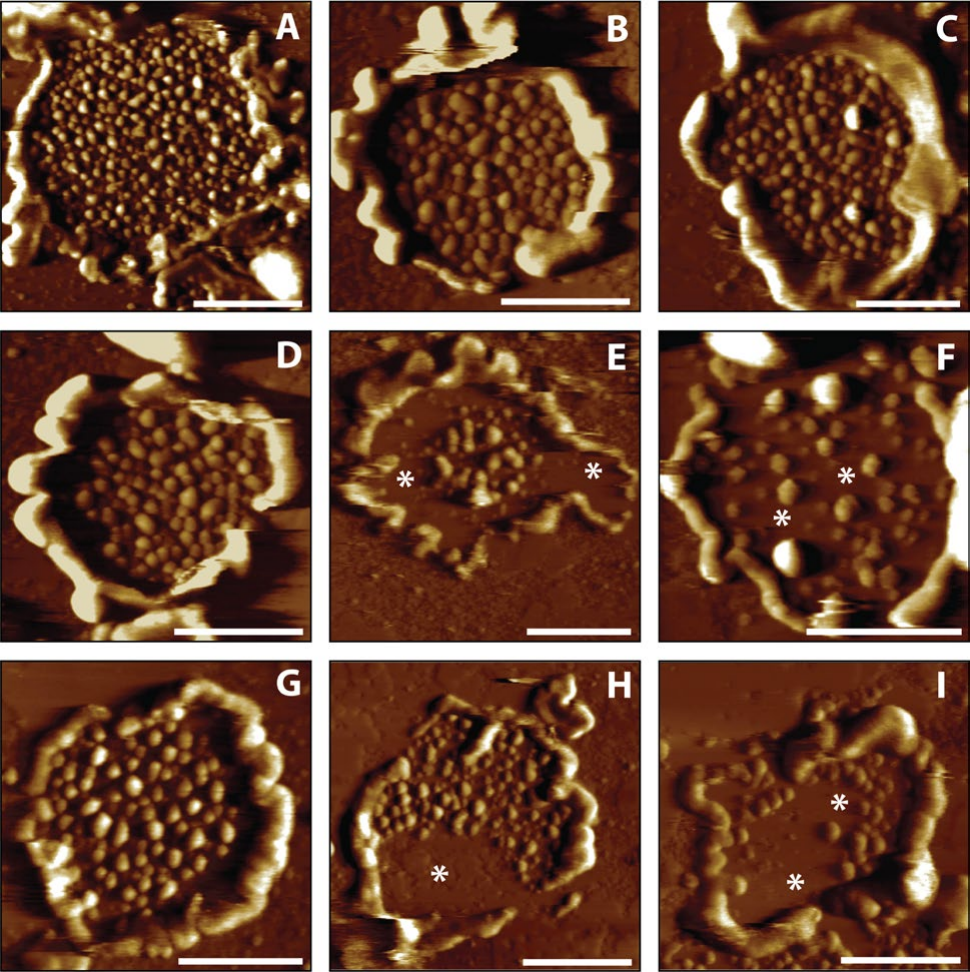
Representative AFM images of ROS disc membranes isolated from WT and *Prcd-*KO retina. Representative AFM images of ROS disc membranes isolated from (**A-C**) WT retina at P120, (**D–F**)* Prcd*-KO retina at P30, and (**G-I**)* Prcd*-KO retina at P120. Asterisks indicate large areas without rhodopsin nanodomains. Scale bar = 500 nm.

**Figure 5:**
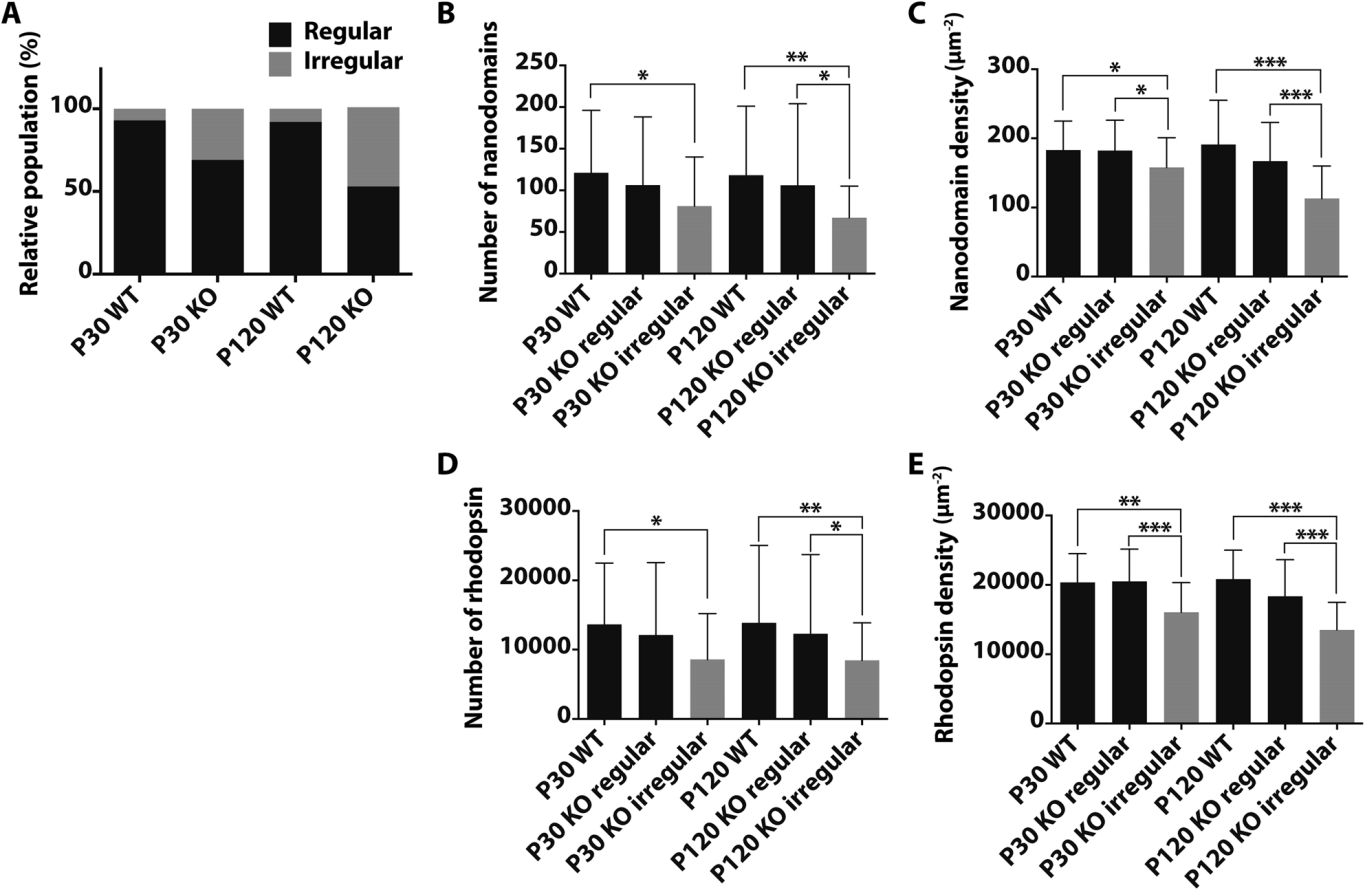
Quantification of ROS disc membrane properties of WT and *Prcd-KO* mice. (**A**) Relative population of regular (black) and irregular discs (grey). (**B**) Number of rhodopsin nanodomains packed into a ROS disc membrane. (**C**) Density of rhodopsin nanodomains within a ROS disc membrane. (**D**) Number of rhodopsin molecules packed into a ROS disc membrane. (**E**) Density of rhodopsin molecules within a ROS disc membrane. The number of ROS disc membranes analyzed are as follows: P30 WT, n = 48; P30 KO regular, n = 61; P30 KO irregular, n = 27; P120 WT, n = 47; P120 KO regular, n = 53; P120 KO irregular, n = 48. Mean values are reported with the standard deviation (unpaired, two-tailed t-test; *p ≤ 0.05, **p ≤ 0.01, ***p ≤ .00001).

To investigate the interplay between rhodopsin and the observed defects in the *Prcd-*KO ROS ultrastructure, we evaluated ROS disc membranes isolated from *Prcd*-KO mice and age matched WT controls by atomic force microscopy (AFM). Previous studies have successfully used AFM to visualize rhodopsin nanodomains in the disc membranes^39^. Rhodopsin nanodomains are formed by oligomers of rhodopsin of various size and are not an artifact of phase separation of lipids or those related to the AFM procedure^28,30,40^. To further our confidence in this detection method, ROS disc membranes from WT mice at P30 and P120 exhibited properties similar to those reported previously^30,32^ (Fig. 4A–C). In these discs, rhodopsin was arranged into nanodomains and the nanodomains were largely densely packed within the disc (Fig. 4A–C). *Prcd*-KO mice at P30 and P120 exhibited some ROS disc membranes which resembled those from WT mice with rhodopsin nanodomains densely packed within the membrane (Fig. 4D,G); however, they also exhibited ROS disc membranes that were irregular. Irregular ROS disc membranes from *Prcd-*KO discs had fewer rhodopsin nanodomains with larger areas devoid of rhodopsin nanodomains (Fig. 4E,F,H, I; asterisks). Irregular ROS disc membranes were also observed for WT mice; however, they were only sporadically observed, representing 6% or less of the ROS disc membranes analyzed. The proportion of regular and irregular ROS disc membranes were quantified for P30 and P120 *Prcd-*KO mice. In P30 KO mice, about one-third of the ROS disc membranes were irregular, and this increased for P120 mice, where about half of the ROS disc membranes were irregular (Fig. 5A, Table 1). Thus, the proportion of irregular ROS disc membranes increased with age for *Prcd-*KO mice. Since ROS discs from photoreceptor cells are heterogeneous, quantitative analysis is required^32^. The number of rhodopsin contained within a ROS disc membrane was estimated from the size of nanodomains and the density of rhodopsin was computed presuming single rhodopsin molecules homogenously distribute throughout the area of the lamellar region of the discs^39^. The number and density of rhodopsin nanodomains and molecules of rhodopsin in the irregular ROS disc membranes of both P30 and P120 mice were lower compared to that of regular ROS disc membranes (Fig. 5B–E). The regular ROS disc membranes of both P30 and P120 *Prcd-*KO mice contained a similar number and density of rhodopsin as that of WT ROS disc membranes (Table 2). Altogether, these data demonstrate that *Prcd-*KO mice have an impaired ability to form proper ROS disc membranes with sufficient rhodopsin number and packaging density.

**Table 1:** Relative population of regular and irregular discs observed in WT and PRCD KO mice across ages (P30 and P120).

Mice	Regular disc (%)	Irregular disc (%)
WT (P30)	93	7
WT (P120)	92	8
*Prcd-*KO (P30)	69	31
*Prcd-*KO (P120)	52	48

**Table 2:** ROS disc properties of 129/SV-E wild-type (WT) and *Prcd-*KO mice across ages (P30 and P120).

Parameter value	Nanodomains of rhodopsin		Rhodopsin molecules	
	Number	Density (µm^2^)	Number	Density (µm^2^)
P30 WT (n = 42)	121 ± 75	183 ± 42	13,642 ± 8848	20,346 ± 4171
P120 WT (n = 47)	118 ± 83	191 ± 64	13,857 ± 11,177	20,828 ± 4171
P30 *Prcd*-KO, regular (n = 61)	106 ± 82	182 ± 44	12,089 ± 10,465	20,494 ± 4658
P30 *Prcd*-KO, irregular (n = 27)	81 ± 59	158 ± 43	8582 ± 6621	16,059 ± 4276
P120 *Prcd*-KO, regular (n = 53)	106 ± 98	167 ± 56	12,290 ± 11,434	18,345 ± 5297
P120 *Prcd*-KO, irregular (n = 48)	67 ± 38	113 ± 47	8454 ± 5417	13,497 ± 3991

## Discussion

In the current study, we sought to further understand the role of PRCD, a small disc-resident protein, in photoreceptor disc morphogenesis. Mutations in PRCD are associated with dramatic misalignment of disc membranes and disorganization of the OS, followed by slow retinal degeneration. The most common PRCD mutation (C2Y) has been extensively characterized in several dog breeds^16^. Recent studies in additional mouse models have shown that high fidelity disc morphogenesis is compromised when PRCD is not present^34,35^. However, the precise role PRCD plays in this process remains unclear. In this study, we demonstrate that loss of PRCD disrupts rhodopsin packaging into ROS disc membranes, leading to defects in visual function and compromised OS structure, phenotypes consistent with clinical findings in humans and dogs with a PRCD-C2Y mutation^10^.

Previous studies in canines demonstrate that PRCD-associated disease is heterogeneous, with spatial and temporal differences in disease onset and progression between dog breeds^14,15,41^ In these models, it is reported that changes in ERG response correspond to the presence of morphological defects in affected animals^16^. In our *Prcd*-KO mouse model, retina appear to develop normally, exhibiting proper lamination. Our ERG studies demonstrate that loss of PRCD impairs mass rod photoreceptor response in bright light conditions as early as P30. At P30, ultrastructural analysis reveals *Prcd-*KO rod photoreceptors with defects in OS morphology, including focal misalignment of discs and imperfect disc stacking. As mice age, ERG response continues to decline and more pronounced morphological defects are observed. Importantly, all tested disc-resident and phototransduction proteins appear normal in *Prcd*-KO retina at P30, suggesting the observed reduction in *Prcd-*KO rod response at this age is due to morphological defects rather than loss of crucial phototransduction proteins^34^.

Similar to two recently published mouse models lacking PRCD, photoreceptor degeneration in our *Prcd-*KO mice occurs well after disease onset, as we only see significant degeneration after P120. However, it seems that disease onset occurs earlier in our mouse model compared to others. In one model, Spencer et al. report that single cell recording of rods shows no differences in visual response compared to WT at P40-P45. Additionally, *Prcd*^−/−^ rods appear relatively normal compared to WT at 2 months, with only a few deformed ROS^34^. In another model, Allon et al. report that mass rod response was not reduced until after 20 weeks of age and do not mention disorganized disc membranes until 30 weeks^35^. We believe these discrepancies can be explained by mouse strain differences and genetic heterogeneity, as our *Prcd-*KO model is in a 129/SV-E background and the other two are in a C57BL/6J background. If the characterized correlation between ERG response and morphology in canines with a PRCD-C2Y mutation holds true in *Prcd-*KO animals, it explains why we see reductions in ERG response at P30 when we also observe morphological defects. Additionally, it may explain why ERG response is not affected in these other knockout models, when ROS appear normal.

These models also show that retina lacking PRCD develop an abundance of extracellular vesicles (EV) containing rhodopsin, which phagocytic microglia cannot manage to clear properly^34,35^. Spencer et al. demonstrates that discs lacking PRCD do not form properly and that prior to flattening, membrane bulges containing rhodopsin separate into EV in the interphotoreceptor space^34^. We were able to identify several examples of EV in our model, but they often appear in the same areas as fixation artifacts and were also observed in WT retina. These newly formed EV are likely labile structures which require cardiac perfusion to retain their ultrastructure. It is important to note that the presence of vesicular profiles is also a hallmark of PRCD-associated disease in miniature poodles^14^. However, these vesicular profiles were reported to be missing or sparsely distributed in retina of English cocker spaniels with PRCD-C2Y mutations^16^. Therefore, while the traditional TEM fixation protocols used in this study may not have captured these vesicles properly, it is also possible that formation of these vesicles is just another phenotypic variation that can be attributed to genetic differences in mouse strain, as demonstrated in canine models. We plan to investigate this possibility in our future studies. In general, the variable phenotypes observed in canines and mice with PRCD-associated disease underscores the importance of continued study of this protein in multiple model systems.

In contrast to other published studies, we furthered our analysis of *Prcd-*KO disc membranes using AFM. PRCD is identified as one of 11 proteins exclusively present in the disc membrane and has been shown to interact with rhodopsin^7,18^. As mentioned, rhodopsin is organized into nanoscale domains and AFM has been extensively used to visualize these nanodomains in various studies^28,30,32,42,43^. Rhodopsin protein is reduced by half in Rho+/− retina and AFM analysis of Rho+/− discs demonstrates that although disc size decreases in response to availability of rhodopsin, at 6 weeks of age, Rho+/− disc membranes contain a density of rhodopsin comparable to WT^22,32^. With an average density of about 20,000 μm^2^ in murine discs, rhodopsin is likely maintained at this density in order for rods to retain high sensitivity to light^23,30,33,39,44,45^. In fact, high rhodopsin packaging density appears very important for proper visual function, as mice housed in constant dark display increased rhodopsin packing density within ROS disc membranes and show improved visual function compared to mice housed in cyclic light conditions^33^. In contrast to tight regulation of rhodopsin packaging density, our *Prcd*-KO mice demonstrate a population (~ 33%) of “irregular” discs at P30 which contain both fewer molecules of rhodopsin and decreased density of rhodopsin in comparison to WT “regular” discs. By P120, the relative proportion of these irregular discs increases to ~ 50%, corresponding with worsening retinal defects as assessed by ERG and TEM. Thus, if there do exist mechanisms to maintain consistent rhodopsin packaging density in ROS disc membranes, our data suggest that PRCD may be involved in regulation of this process.

Although rhodopsin levels and availability appear relatively normal in *Prcd*-KO retina at P30, there is an apparent inability for rod photoreceptor neurons lacking PRCD to maintain the proper density of rhodopsin within ROS disc membranes. Furthermore, the presence of these irregular discs strongly suggests a possible explanation for the observed reduction in *Prcd-*KO rod ERG response and ROS ultrastructural defects at P30 and subsequent ages. By P120, we detect an approximate 20.4% reduction in total rhodopsin protein. We speculate that this decrease is related to the disrupted incorporation of rhodopsin into these aforementioned irregular discs.

In all PRCD knockout models to date, cones appear unaffected until rods degenerate. As cones rely on opsins other than rhodopsin for phototransduction, the specific role for PRCD in rod photoreceptors through packaging of rhodopsin into disc membranes is compelling. Considering PRCD’s small size, one hypothesis is that PRCD works by anchoring nascent discs in place to ensure that the proper density of rhodopsin is packed into each disc before flattening and enclosure. Investigation of this possibility, as well as determining why *Prcd-*KO rods are still able to form a population of phenotypically normal discs, are topics of future study.

Altogether, our data provide further insight into PRCD’s involvement in proper rhodopsin packaging density in ROS disc membranes, a process which is crucial for OS maintenance and disc morphogenesis.

## Methods

### Generation of *Prcd* knockout model

*Prcd*-KO mice were generated using CRISPR/Cas9 genome editing at the transgenic core facility at West Virginia University. Small guide RNA (sgRNA) were designed as described in earlier studies^46,47^. Two sgRNA target sequences were created for exon 1 of *Prcd*, both upstream (CCGTAGATTTACCAACCGAG*TGG*) and downstream (CCACTCGGTTGGTAAATCTA*CGG*) of the 5′-NGG “PAM” (protospacer adjacent motif). These sequences were annealed and ligated into a pX330 vector (pSpCas9). Using PCR amplification, a T7 promoter (TTAATACGACTCACTATAGGG) was added to the sgRNA template and purified using an RNA purification kit from Ambion. After confirming the specificity and efficacy of the sgRNA/Cas9 cutting by *in-vitro* assays, both sgRNA (17 ng/μl) and Cas9 (34 ng/μl) mRNA (Invitrogen) were injected into the pronuclei of FVB blastocysts. The correctly targeted founder mice were identified by sequencing and backcrossed with 129/SV-E mice (Charles River Laboratories) to eliminate the *rd1* allele, which naturally occurs in FVB mice. Furthermore, *Prcd-*KO mice were confirmed to lack the *rd8* mutation and were extensively backcrossed for more than 6 generations with 129/SV-E mice to rule out “off-target” effects sometimes associated with CRISPR/Cas9 gene editing. Genotyping was performed by PCR amplification followed by sequencing. All experimental procedures involving animals in this study were approved and conducted in strict accordance with relevant guidelines and regulations by the Institutional Animal Care and Use Committee at West Virginia University.

### Electroretinography

Prior to measuring light dependent electrical response, mice were dark-adapted overnight before testing and ERGs were performed under dim red light. Animals were anesthetized with isoflurane (5% in 2.5% oxygen) and eyes were dilated (1:1 Phenylephrine : Tropicamide) for 10 min and placed on a stage heated to 37 °C with a nose cone supplying isoflurane (1.5% in 2.5% oxygen) for testing. After lubrication with GenTeal gel (0.3% Hypromellose), silver electrodes were placed above the cornea to measure ERG response. A reference electrode was placed between the ears on top of the animal’s head. ERG recordings were conducted using the UTAS Visual Diagnostic System with BigShot Ganzfeld, UBA-4200 amplifier and interface, and EMWIN software (version 9.0.0, LKC Technologies, Gaithersburg, MD, USA). Scotopic (rod) ERGs were performed in the dark with flashes of white light at increasing intensities. Photopic (cone) responses were performed with flashes of increasing intensities of white light with a 30 cd/m^2^ white background light for 10 min to saturate rods.

### Immunoblotting

To evaluate protein expression by immunoblot, two retinas were homogenized by sonication (Microson Ultrasonic cell disruptor, 3 pulses 5 s at power setting 10) in 200 μl of urea sample buffer (USB) containing 6 M, 4% SDS, and 125 mM Tris–HCl, pH 6.8 in a 1.5-ml microcentrifuge tube on ice. Protein concentration was determined using a NanoDrop spectrophotometer (ND-1000, Thermo Fisher Scientific). Samples were normalized to 5 mg/ml with additional USB and 5% 2-mercaptoethanol and bromophenol blue were added prior to SDS-PAGE. Equal concentration of each sample was loaded and separated by SDS-PAGE in a 4–15% tris–glycine precast gel (4–20% Mini-PROTEAN TGX, Bio-Rad, Hercules, CA, USA) and subsequently transferred to an Immobilon-FL PVDF membrane (Immobilon-FL, Millipore, Burlington, MA, USA). After blocking membranes with blocking buffer (Odyssey Blocking Buffer; LI-COR Biosciences, Lincoln, NE, USA) for 1 h at room temperature (RT) and incubated with primary antibodies for 2 h at RT or overnight at 4 °C on a bidirectional rotator. After primary antibody incubation (see Table 3), immunoblots were washed three times in 1X PBST (1X PBS/0.1% Tween-20) for 5 min each at RT and incubated with secondary antibody, goat anti-rabbit Alexa Fluor 680, goat anti-mouse DyLight 800 or donkey anti-sheep Alexa Fluor 680 (Thermo Fisher Scientific) for 30 min at RT. After washing three additional times in 1X PBST, membranes were scanned using an Odyssey Infrared Imaging System and protein density was measured according to manufacturer’s instruction (LI-COR Biosciences, Lincoln, NE, USA).

**Table 3:** List of primary antibodies used in this study.

Antibody	Source	Dilution
Rabbit anti-PRCD	Lab generated	1:1000
Mouse anti-Rhodopsin (4D2)	Gift from R. Molday, Univ. British Columbia	1:2000
Mouse anti-GAPDH	Proteintech, 60004-1-Ig	1:10,000
Rabbit anti-PDE6β	Thermo Fisher Scientific, PA 1-722	1:2000
Rabbit anti-GNAT1	Proteintech, 55167-1-AP	1:2000
Rabbit anti-ROM1	Gift from G. Travis, UCLA	1:2000
Rabbit anti-PRPH2	Gift from G. Travis, UCLA	1:2000
Sheep anti-RGS9	Gift from M. Sokolov, WVU	1:5000
Rabbit anti-GC-1	Gift from V. Ramamurthy, WVU	1:2000

### Immunohistochemistry

Whole eyes from P30, P120, and P250 were enucleated, immersed, and fixed in Excalibur’s Z-fix. Embedding, sectioning, and hematoxylin and eosin (H&E) staining was carried out on WT and *Prcd*-KO eyes by Excalibur Pathology, Inc. (Norman, OK, USA). Images were collected using a Nikon C2 confocal microscope. Five points were chosen to count the number of photoreceptor nuclei in the outer nuclear layer (every 350 μm) above and below the optic nerve. Counts were completed for three independent eyes (n = 3) and averaged to determine the amount of photoreceptor degeneration at each age. Images were processed using ImageJ software along with the Bio-Formats plugin^48,49^.

### Immunofluorescent staining

After enucleation, mouse eyes were prepared as described previously^17^. Using a Leica CM1850 Cyrostat, 16 μM cross-sections were cut and placed on Superfrost plus slides (Fisher Scientific). Slides were washed with 1X PBS to hydrate tissue and to remove OCT. To prevent non-specific antibody labeling, slides were blocked for 1 h. After blocking, primary antibodies were added at a 1:1000 dilution and incubated overnight at 4 °C (see Table 3). After primary antibody incubation, sections were washed in 1X PBST (1X PBS/0.1% Triton X-100) three times and incubated with the nuclear stain DAPI (1:5,000; Molecular Probes) and secondary antibody (Thermo Fisher Scientific goat anti-mouse Alexa Fluor-488 or goat anti-rabbit Alexa Fluor-568) at a 1:1,000 dilution for 1 h at RT. Slides were mounted with Prolong Gold antifade reagent (Thermo Fisher Scientific) and coverslipped (1 mm). Slides were imaged using a Nikon C2 confocal microscope. Images were processed using ImageJ software along with the Bio-Formats plugin^48,49^.

### Transmission electron microscopy

Enucleated eyes were placed in freshly prepared buffer containing 2% paraformaldehyde, 2.5% glutaraldehyde, and 0.1 M cacodylate buffer, pH 7.5. After 30 min of fixation, cornea and lens were removed, and eyecups were fixed for an additional 48 h at RT with constant rotation on a nutator. Dissection, embedding, and transmission electron microscopy were performed as described previously^50^.

### Rod outer segment and disc membrane isolation

Retina from 15 age-matched WT (129/SV-E) and *Prcd*-KO mice were collected. Retinal tissues were suspended in 300 μl of 8% (vol/vol) Optiprep (Sigma-Aldrich, St. Louis, MO, USA) in Ringer’s buffer (10 mM HEPES, 130 mM NaCl, 3.6 mM KCl, 2.4 mM MgCl_2_, 1.2 mM CaCl_2_, and 0.02 mM EDTA) and vortexed for 1 min. Next, samples were centrifuged at 238 × *g* for 1 min at 4ºC and the supernatant was collected into a clean tube. This was repeated four additional times and the subsequent supernatant was pooled and placed on top of a 10–30% Optiprep in Ringer’s buffer gradient. The gradient containing the sample was then centrifuged at 26,500 × *g* for 50 min at 4ºC with no brakes (Beckman Coulter Optima LE-80 K; SW-41Ti). A dense band approximately two-thirds from the top of the gradient was collected and diluted fourfold in Ringer’s buffer. After a 3 min centrifugation at 627 × *g* at 4ºC, the supernatant was next transferred to a high-speed centrifuge tube and centrifuged at 26,500 × *g* for 30 min at 4ºC (Beckman Coulter Optima TLX; rotor-TLA55). The resulting pellet was then resuspended in 2 mM Tris–HCl and used for AFM studies.

### Atomic force microscopy

All experimental procedures were conducted under dim red light conditions to avoid photobleaching. ROS disc membranes were diluted in Ringer's buffer (10 mM HEPES, 130 mM NaCl, 3.6 mM KCl, 2.4 mM MgCl_2_, 1.2 mM CaCl_2_, and 0.02 mM EDTA, pH 7.4), immobilized on freshly cleaved mica and imaged by AFM in imaging buffer (20 mM Tris, 150 mM KCl, 25 mM MgCl_2_, pH 7.8), as described previously^32^. Contact mode AFM imaging was performed using Bruker Multimode II atomic force microscope equipped with an E scanner (13 μm scan size) and silicon nitride cantilevers with a nominal spring constant of 0.06 N/m (DNP-S, Bruker, Santa Barbara, CA). Deflection images were analyzed using SPIP (version 6.5, Image Metrology A/S, Hørsholm, Denmark) to determine the number and density of rhodopsin, as described previously^32,39^. Graphs and statistical analyses were done using Prism 7 (GraphPad Software, San Diego, CA).

### Rhodopsin measurement

The amount of rhodopsin (picomoles per retina) was assessed using a modified protocol^37,38^. The concentration of rhodopsin was determined by measuring the difference in absorbance using a spectrophotometer before and after bleaching of the sample with a known light intensity. Individual retina were dissected in the dark under dim red light and placed in 200 μl of water containing 10% n-octyl-β-d-glucopyranoside and 50 μl of 200 mM hydroxylamine, pH 7.5. After sonication, the sample was centrifuged at 14,000 rpm in a tabletop centrifuge for 30 s and 150 μl of sample was measured by spectrophotometer before and after light exposure. Absorbance and a molar extinction coefficient of 40,500 were used to calculate the concentration of rhodopsin per retina^37,38^.

### Statistical analysis

Data are expressed as means ± S.E.M., unless otherwise indicated. The differences between littermate or age matched control (WT) and experimental animals (*Prcd*-KO) were analyzed with a two-tailed student *t* test (online version, https://www.socscistatistics.com/).

## Supplementary information

Supplementary Information.

## References

[1] Burns ME, Arshavsky VY (2005). Beyond counting photons: trials and trends in vertebrate visual transduction. Neuron.

[2] Goldberg, A. F. X. in *International Review of Cytology* Vol. 253, 131–175 (Academic Press, London, 2006). 10.1016/S0074-7696(06)53004-9 17098056

[3] Sung C-H, Chuang J-Z (2010). The cell biology of vision. J. Cell Biol..

[4] Goldberg AF, Moritz OL, Williams DS (2016). Molecular basis for photoreceptor outer segment architecture. Prog. Retin. Eye Res..

[5] Palczewski K (2012). Chemistry and biology of vision. J. Biol. Chem..

[6] Wensel TG (2016). Structural and molecular bases of rod photoreceptor morphogenesis and disease. Prog. Retin. Eye Res..

[7] Skiba NP (2013). Proteomic identification of unique photoreceptor disc components reveals the presence of PRCD, a protein linked to retinal degeneration. J. Proteome Res..

[8] Downs LM, Hitti R, Pregnolato S, Mellersh CS (2014). Genetic screening for PRA-associated mutations in multiple dog breeds shows that PRA is heterogeneous within and between breeds. Vet. Ophthalmol..

[9] Goldstein O (2006). Linkage disequilibrium mapping in domestic dog breeds narrows the progressive rod-cone degeneration interval and identifies ancestral disease-transmitting chromosome. Genomics.

[10] Zangerl B (2006). Identical mutation in a novel retinal gene causes progressive rod-cone degeneration in dogs and retinitis pigmentosa in humans. Genomics.

[11] Hartong DT, Berson EL, Dryja TP (2006). Retinitis pigmentosa. The Lancet.

[12] Verbakel SK (2018). Non-syndromic retinitis pigmentosa. Prog. Retin. Eye Res..

[13] Parry HB (1953). Degenerations of the Dog Retina. Br. J. Ophthalmol..

[14] Aguirre G, Alligood J, O’Brien P, Buyukmihci N (1982). Pathogenesis of progressive rod-cone degeneration in miniature poodles. Invest. Ophthalmol. Vis. Sci..

[15] Aguirre G, O'Brien P (1986). Morphological and biochemical studies of canine progressive rod-cone degeneration. 3H-fucose autoradiography. Investig. Ophthal. Vis. Sci..

[16] Aguirre GD, Acland GM (1988). Variation in retinal degeneration phenotype inherited at the prcd locus. Exp. Eye Res..

[17] Murphy J, Kolandaivelu S (2016). Palmitoylation of progressive rod-cone degeneration (PRCD) regulates protein stability and localization. J. Biol. Chem..

[18] Spencer WJ (2016). Progressive rod-cone degeneration (PRCD) protein requires N-terminal S-acylation and rhodopsin binding for photoreceptor outer segment localization and maintaining intracellular stability. Biochemistry.

[19] Papermaster DS, Dreyer WJ (1974). Rhodopsin content in the outer segment membranes of bovine and frog retinal rods. Biochemistry.

[20] Palczewski K (2006). G protein-coupled receptor rhodopsin. Annu. Rev. Biochem..

[21] Chakraborty D, Conley SM, Al-Ubaidi MR, Naash MI (2014). Initiation of rod outer segment disc formation requires RDS. PLoS ONE.

[22] Liang Y (2004). Rhodopsin signaling and organization in heterozygote rhodopsin knockout mice. J. Biol. Chem..

[23] Makino CL (2012). Rhodopsin expression level affects rod outer segment morphology and photoresponse kinetics. PLoS ONE.

[24] Sakami S, Kolesnikov AV, Kefalov VJ, Palczewski K (2014). P23H opsin knock-in mice reveal a novel step in retinal rod disc morphogenesis. Hum. Mol. Genet..

[25] Wen XH (2009). Overexpression of rhodopsin alters the structure and photoresponse of rod photoreceptors. Biophys. J..

[26] Humphries MM (1997). Retinopathy induced in mice by targeted disruption of the rhodopsin gene. Nat. Genet..

[27] Lem J (1999). Morphological, physiological, and biochemical changes in rhodopsin knockout mice. Proc. Natl. Acad. Sci..

[28] Liang Y (2003). Organization of the G protein-coupled receptors rhodopsin and opsin in native membranes. J. Biol. Chem..

[29] Fotiadis D (2003). Rhodopsin dimers in native disc membranes. Nature.

[30] Rakshit T, Senapati S, Sinha S, Whited AM, Park PSH (2015). Rhodopsin forms nanodomains in rod outer segment disc membranes of the cold-blooded *Xenopus laevis*. PLoS ONE.

[31] Whited AM, Park PS (1838). Atomic force microscopy: a multifaceted tool to study membrane proteins and their interactions with ligands. Biochim. Biophys. Acta.

[32] Rakshit T, Park PS (2015). Impact of reduced rhodopsin expression on the structure of rod outer segment disc membranes. Biochemistry.

[33] Rakshit T (1864). Adaptations in rod outer segment disc membranes in response to environmental lighting conditions. Biochim. Biophys. Acta Mol. Cell Res..

[34] Spencer WJ (2019). PRCD is essential for high-fidelity photoreceptor disc formation. Proc. Natl. Acad. Sci. U S A.

[35] Allon G (2019). PRCD is concentrated at the base of photoreceptor outer segments and is involved in outer segment disc formation. Hum. Mol. Genet..

[36] Pinto LH, Invergo B, Shimomura K, Takahashi JS, Troy JB (2007). Interpretation of the mouse electroretinogram. Doc. Ophthalmol..

[37] Sokolov M (2002). Massive light-driven translocation of transducin between the two major compartments of rod cells: a novel mechanism of light adaptation. Neuron.

[38] Strissel KJ, Sokolov M, Trieu LH, Arshavsky VY (2006). Arrestin translocation is induced at a critical threshold of visual signaling and is superstoichiometric to bleached rhodopsin. J. Neurosci..

[39] Senapati S, Park PS (1886). Investigating the nanodomain organization of rhodopsin in native membranes by atomic force microscopy. Methods Mol. Biol..

[40] Gunkel M (2015). Higher-order architecture of rhodopsin in intact photoreceptors and its implication for phototransduction kinetics. Structure.

[41] Aguirre GD, Rubin LF (1972). Progressive retinal atrophy in the Miniature Poodle: an electrophysiologic study. J. Am. Vet. Med. Assoc..

[42] Buzhynskyy N, Salesse C, Scheuring S (2011). Rhodopsin is spatially heterogeneously distributed in rod outer segment disk membranes. J. Mol. Recognit..

[43] Whited AM, Park PSH (1848). Nanodomain organization of rhodopsin in native human and murine rod outer segment disc membranes. Biochem. Biophys. Acta..

[44] Nickell S, Park PS, Baumeister W, Palczewski K (2007). Three-dimensional architecture of murine rod outer segments determined by cryoelectron tomography. J. Cell Biol..

[45] Calvert PD (2001). Membrane protein diffusion sets the speed of rod phototransduction. Nature.

[46] Cong L (2013). Multiplex genome engineering using CRISPR/Cas systems. Science.

[47] Ran FA (2013). Genome engineering using the CRISPR-Cas9 system. Nat. Protoc..

[48] Schneider CA, Rasband WS, Eliceiri KW (2012). NIH Image to ImageJ: 25 years of image analysis. Nat. Methods.

[49] Linkert M (2010). Metadata matters: access to image data in the real world. J. Cell Biol..

[50] Goldberg AFX (2007). An Intramembrane Glutamic Acid Governs Peripherin/rds Function for Photoreceptor Disk Morphogenesis. Invest. Ophthalmol. Vis. Sci..

