# Supplementary material for: Loss of PRCD alters number and packaging density of rhodopsin in rod photoreceptor disc membranes
Source: Sci Rep. 2020 Oct 21;10:17885. doi: 10.1038/s41598-020-74628-2 (PMC7577997; doi:10.1038/s41598-020-74628-2)
Supplement: Supplementary file 1 — Supplementary Information. [file 41598_2020_74628_MOESM1_ESM.pdf]

Supplementary Data

Title: Loss of PRCD alters number and packaging density of rhodopsin in rod photoreceptor disc membranes

Authors: Emily R. Sechrest[1,2], Joseph Murphy[2,3], Subhadip Senapati[4], Andrew F.X. Goldberg[5], Paul S.-H. Park[4], Saravanan Kolandaivelu[2,3,*]

Affiliations:

[1]Department of Pharmaceutical Sciences, [2]Department of Ophthalmology and Visual Sciences, [3]Department of Biochemistry, West Virginia University, Morgantown, WV 26506, USA.

[4]Department of Ophthalmology and Visual Sciences, Case Western Reserve University, Cleveland, OH 44106, USA.

[5]Eye Research Institute, Oakland University, Rochester, MI 48309, USA.

***Corresponding author:** Saravanan Kolandaivelu, Departments of Ophthalmology and Visual Sciences and Biochemistry; Eye Institute, One Medical Center Drive, West Virginia University, Morgantown, WV 26506-9193, USA. Email: kolandaivelus@wvumedicine.org; Tel: 304 598 5484; Fax: 304 598 6928.

Supplementary methods

RNA extraction, cDNA synthesis, and quantitative RT-PCR

Total RNA from P30 and P120 retina from WT and *Prcd*-KO animals was purified with TRIzol reagent (Thermo Fisher Scientific) according to manufacturer's protocols. RNA concentration was evaluated using a NanoDrop spectrophotometer (ND-1000, Thermo Fisher Scientific). First strand cDNA synthesis was carried out using the RevertAid First Strand cDNA Synthesis Kit (Thermo Fisher Scientific) using Oligo (dT)18 primer and 2.5 μg of purified total RNA per 20 μl of reaction volume. Each cDNA was diluted 10-fold with nuclease-free water before qRT-PCR. The qRT-PCR reactions were prepared using qPCR Brilliant II SYBR mastermix with ROX (Agilent), 150 nM of each primer, and cDNA from reverse transcription. To prime the cDNA synthesis, the reactions were performed according to the manufacturing instructions using a Stratagene Mx3000P real-time PCR system. Data was acquired using the MxPro QPCR software (Agilent). Each sample (n = 3) was run in triplicate and averaged to produce a single data point. Rhodopsin mRNA levels were analyzed using the primers SK254F (GAATCACGCTATCATGGGTGTGG) and SK255R (ATGACAAAGGATTCGTTGTTGACC). Relative rhodopsin gene expression was evaluated by normalization to the levels of the reference genes *Hmbs* (hydroxymethylbilane synthase) and *Ywhaz* (tyrosine 3-monooxygenase/tryptophan 5-monooxygenase activation protein, zeta polypeptide). *Hmbs* levels were quantified using the primers SK258F (GTTTACCAAGGAGCTAGAAAACGC) and SK259R (GTGAAAGACAACAGCATCACAAGG). *Ywhaz* transcript was amplified using the primers SK260F (GTTGTAGGAGCCCGTAGGTCATCG) and SK261R (GCTTTCTGGTTGCGAAGCATTGGG).

```
                         1        10        20        30        40        50
(a) PRCD WT - 129/SV-E   MCTTLFLFSLAMLWRRRFTNRVEPEPSRVDGTVVGSGSDTDLQSTGREKGPVK
(b) PRCD Δ1bp - #1738    MCTTLFLFSLAMLWRRRFTNEWNQSPAEWTGQSWAAAQTQTFNLPAGRKDL*
(c) PRCD Δ14bp - #1734   MCTTLFLFSLAMLWRRRFTRAQQSGRDSRGQRGQRLRHRPSIYRQGERTCEV
(d) PRCD Δ71bp - #1748   ----------------SGTRAQQSGRDSRGQRGQRLRHRPSIYRQGERTCEV
```

Supplementary Figure S1. Depiction of mutations in *Prcd*-KO mice by CRISPR/Cas9 genome editing. The amino acid sequence of (a) WT PRCD protein from 129/SV-E mice compared to *Prcd*-KO founder lines (b) 1738 with a 1 bp deletion, (c) 1734 with a 14 bp deletion, and (d) 1748 with a 71 bp deletion. Red represents the frameshift mutation, green represents a start codon, and asterisks represent an early stop codon.

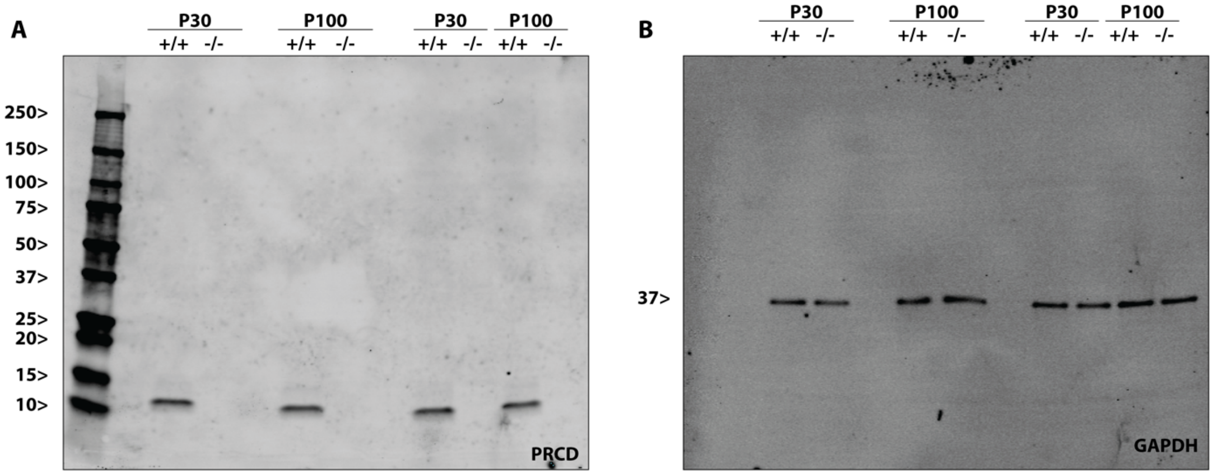

Supplementary Figure S2. Uncropped data corresponding to Figure 1B. Validation of *Prcd*-KO animal model. Lysate from both *Prcd*-KO and WT littermate control retina at P30 and P100 were subjected to western blot analysis and probed for **(A)** PRCD, progressive rod-cone degeneration and **(B)** GAPDH (served as a loading control). Please note that samples were loaded in duplicate.

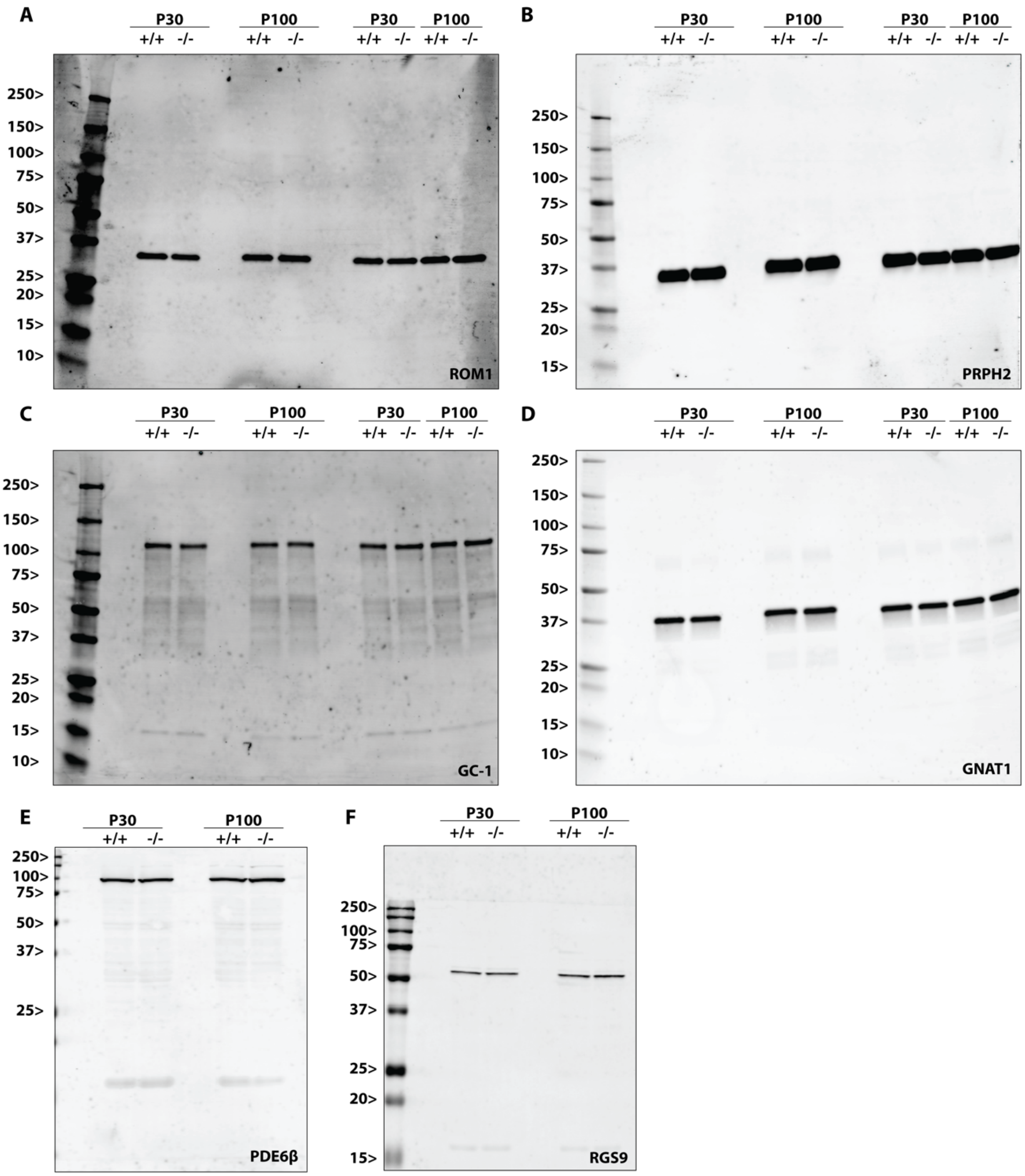

Supplementary Figure S3. Normal levels of major photoreceptor specific proteins in *Prcd*-KO retina. Lysate from both *Prcd*-KO and WT littermate control retina were subjected to western blot analysis. Blots were probed for several phototransduction and OS-disc specific

proteins at P30 and P100 with antibodies against **(A)** ROM1, rod outer segment membrane protein-1, **(B)** PRPH2, peripherin 2, **(C)** GC-1, guanylate cyclase-1, **(D)** GNAT1, rod transducin α, **(E)** PDE6β, phosphodiesterase-6 subunit beta, **(F)** RGS9, regulator of G-protein signaling-9. GAPDH served as a loading control (as shown in Supplementary Fig. S2). Please note that Supplementary Fig. S4A-D were loaded in duplicate.

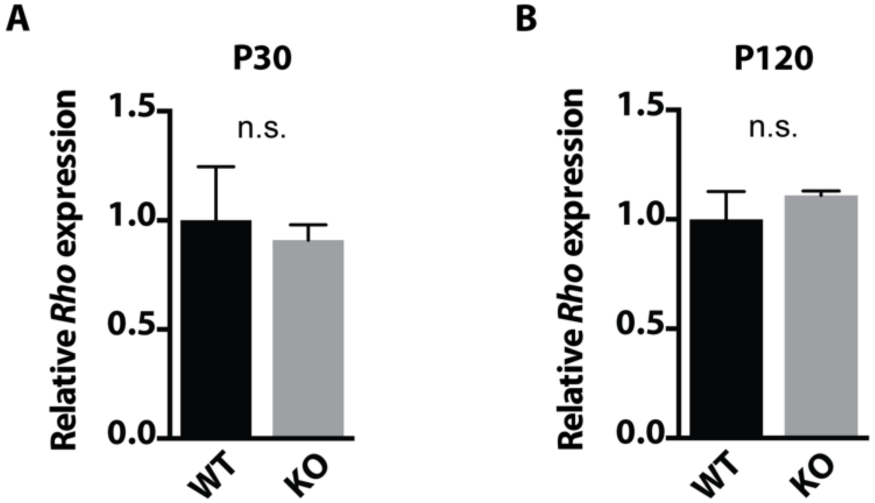

Supplementary Figure S4. No alteration in rhodopsin gene expression at P30 and P120. WT and *Prcd*-KO rhodopsin mRNA levels are comparable at **(A)** P30 and **(B)** P120. *Rho* gene expression was normalized to mRNA levels of the reference genes *Ywhaz* and *Hmbs*. Data are represented as mean relative *Rho* expression (*n*=3, unpaired two-tailed t-test). (*Ymhaz;* tyrosine 3-monooxygenase/tryptophan 5-monooxygenase activation protein, zeta polypeptide, and *Hmbs;* hydroxymethylbilane synthase).

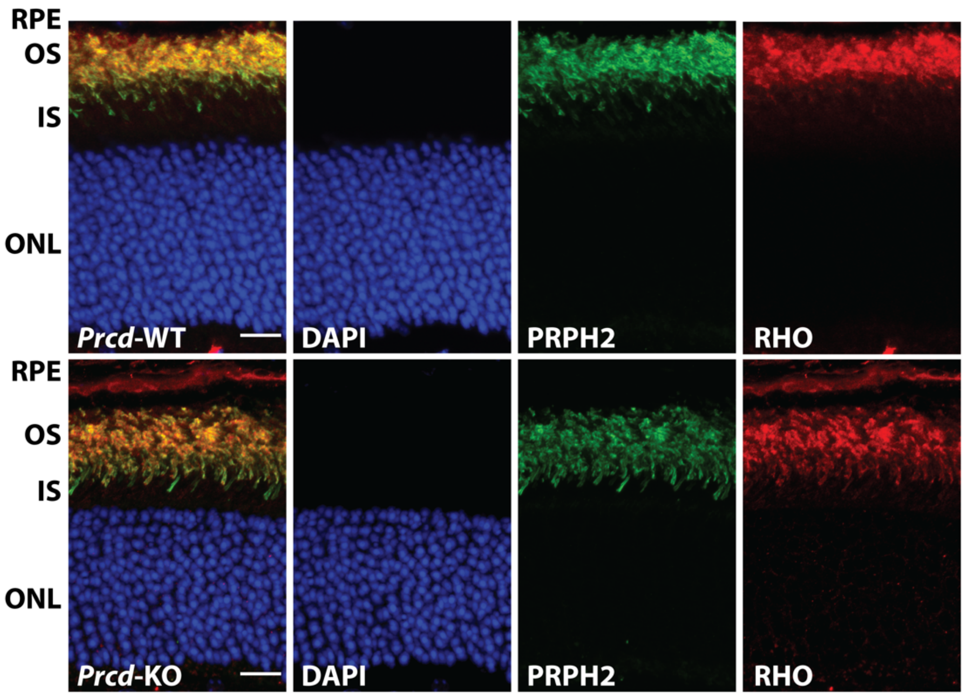

Supplementary Figure S5. Localization of rhodopsin and peripherin-2 in *Prcd*-KO retina at P30. Cross-sections from *Prcd*-KO and WT littermate controls were subjected to immunofluorescent staining with antibodies against the OS disc rim-specific protein PRPH2 (green) and the OS disc lamellae protein RHO (red) at P30. Scale bar = 20 μm

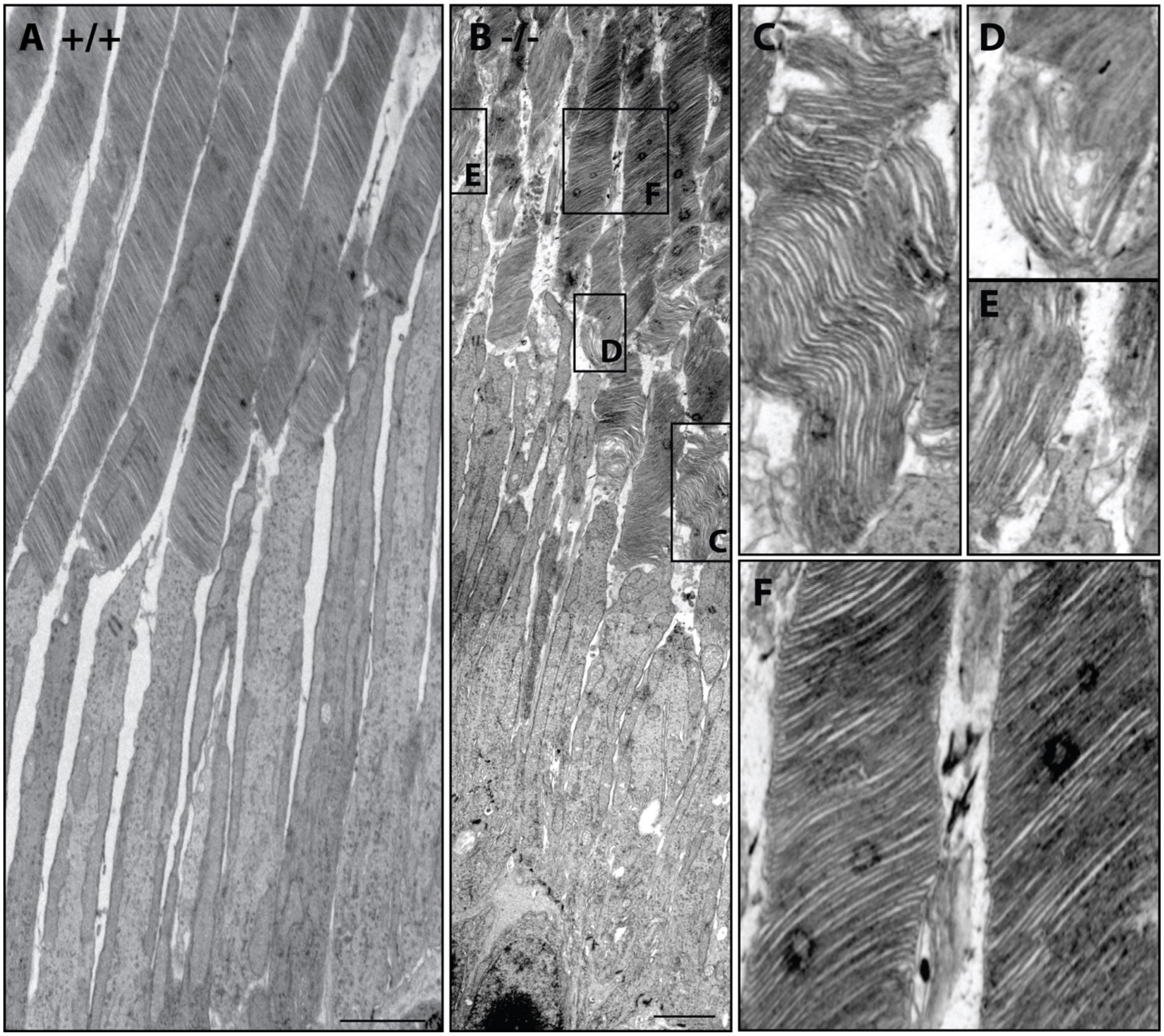

Supplementary Figure S6. Defective photoreceptor OS ultrastructure of *Prcd*-KO retina at P60. (A) Representative TEM images of WT and **(B)** *Prcd*-KO photoreceptor OS cross-sections at P60. (**C-E)** *Prcd*-KO ROS displayed some disorganized disc membranes, vertically aligned stacks of enlarged disc membranes, and disrupted OS with disc membranes aligned in a vertical and perpendicular shape. **(F)** *Prcd*-KO cross sections also revealed many photoreceptors with normal OS structure with properly stacked disc membranes. Inlet boxes enlarged from Supplementary Fig. S6B **(C-F)**. Scale bar = 2 μm.
